# Inhibition of porcine reproductive and respiratory syndrome virus replication in vitro using DNA-based short antisense oligonucleotides

**DOI:** 10.1186/s12917-015-0518-2

**Published:** 2015-08-12

**Authors:** Longlong Zheng, Xiang Li, Lingyun Zhu, Wengui Li, Junlong Bi, Guishu Yang, Gefen Yin, Jianping Liu

**Affiliations:** Department of Veterinary Medicine, College of Animal Science and Technology, Yunnan Agricultural University, Kunming, China; Present address: Wulan Institute for Animal Health, Lingyuan, Chaoyang City, Liaoning province China; Present address: Center for Animal Disease Control and Prevention of Chuxiong City, Chuxiong City, Yunnan province China; Present address: Karolinska Institute, Department of Biosciences and Nutrition, Novum, Huddinge Sweden

**Keywords:** Porcine reproductive and respiratory syndrome virus (PRRSV), Virus replication, Antisense oligonucleotides, Marc-145, Pulmonary alveolar macrophages (PAM)

## Abstract

**Background:**

Porcine reproductive and respiratory syndrome (PRRS) is caused by porcine reproductive and respiratory syndrome virus (PRRSV) and is an economically important disease in swine-producing areas. The objective of this study was to screen for effective antisense oligonucleotides (AS-ONs) which could inhibit PRRSV replication in MARC-145 cells and in pulmonary alveolar macrophages (PAM).

**Results:**

Nine short AS-ON sequences against the well-conserved regions of PRRSV (5′-UTR, NSP9, ORF5 and ORF7) were selected. When MARC-145 cells or PAM were infected with PRRSV followed by transfection with AS-ONs, four AS-ON sequences targeting 5′-UTR, ORF5 or NSP9 were found to be the most effective oligonucleotides in decreasing the cytopathic effect (CPE) induced by PRRSV infection. Quantitative PCR and indirect immunofluorescence staining confirmed that ORF7 levels were significantly reduced both at RNA and protein levels. The PRRSV titration data furthermore indicated that transfection with AS-ON YN8 could reduce the PRRSV titer by 1000-fold compared with controls.

**Conclusion:**

The results presented here indicate that DNA-based antisense oligonucleotides can effectively inhibit PRRSV replication in MARC-145 cells and in PAM. Furthermore, comparing with the reported hit rates (approximately 10-30 %), we achieved a higher success rate (44 %). The strategy we took to design the antisense sequences might be applied to select AS-ONs that more efficiently reduce the expression of target genes.

## Background

Porcine reproductive and respiratory syndrome (PRRS) is one of the most economically significant viral diseases in the swine industry, which is characterized by respiratory disorders in piglets and reproductive failure in sows [1]. This disease is due to the infection by porcine reproductive and respiratory syndrome virus (PRRSV), which belongs to the family *Arteriviridae*, genus *Arterivirus* [2]. PRRSV is an enveloped single-stranded positive-sense RNA virus. The genome of PRRSV is approximately 15 kb in length and consists of nine open reading frames (ORFs) [3]. ORF1a and ORF1b are located at the 5′ end of the genome and encode proteins with replicase and polymerase activities. NSP9 is a putative RNA-dependent RNA polymerase and plays important roles in viral replication [4]. ORFs 2–7 are located at the 3′-end of the genome and encode the structural proteins [2]. ORF5 encodes the GP5 protein, a receptor-binding protein [3, 5] which is a primary antigenic envelope glycoprotein. GP5 is targeted by the cellular immune response and is critical for viral neutralization functioning as. ORF7 encodes the nucleocapsid protein N which is important for the assembly and disassembly of the virion [6]. It is reasonable to speculate that antisense oligonucleotides targeting NSP9, ORF5 and ORF7, as well as 5′UTR, will result in degradation of the viral genome and inhibition of viral production.

Antisense technology is one of the most promising technologies allowing the use of a short complementary oligonucleotide fragment to inhibit the expression of the target mRNA at transcriptional as well as at post-transcriptional levels. Antisense technology has the advantage that it shows high specificity and selectivity for the target gene sequence. Theoretically, antisense molecules could be used to treat any disease that is caused by the expression of a gene, e.g. viral infections, cancer growth, and inflammatory diseases [7–9].

Some highly pathogenic PRRSV strains of the North American type were found spread widely in more than 10 provinces in China and brought about four million fatal cases in 2006 [10]. Therefore, it is imperative to develop effective antiviral strategies to prevent and control this infection. In the interest of exploiting improved methods to control PRRS, we have applied the *in silico* oligo-walk method and *in vitro* biological approaches (cytopathic effect observation, quantitative PCR, virus titer assay and indirect immunofluorescence staining) to screen for protective antisense oligonucleotides that inhibit the replication of PRRSV in MARC-145 cells and in PAM.

## Materials and methods

### Ethics statement

Pigs used in this study did not undergo any manipulation prior to standard industrial slaughter according to the pertinent legislations. For this reason, no specific ethical approval was required. All animal experiments were performed with the approval of the Animal Care Committee of Yunnan Agricultural University, China.

### Virus and cells

The PRRSV field strain YN-1 (GenBank accession number: KJ747052), a highly pathogenic PRRSV belonging to the North American genotype, was isolated in 2008 by our research group from the lungs of an infected pig in Yunnan province (China) during a severe PRRSV outbreak. It is known that the PRRSV can replicate in pulmonary alveolar macrophages (PAM) or in MARC-145 cells, thus both culture systems were applied in this study. The MARC-145 cell line was purchased from the Shanghai Cell Collection, Chinese Academy of Sciences (CAS), and cultured in Dulbecco’s modified Eagle’s medium (DMEM, Invitrogen) supplemented with 10 % heat-inactivated fetal bovine serum (FBS, GIBCO) (pH 7.4), 2 mM L-glutamine, 100 U/ml penicillin and 100 μg/ml streptomycin (Invitrogen). The cultures were maintained in a 5 % CO_2_ humidified incubator at 37 °C. PAMs were obtained by *post mortem* lung lavage of 8-week-old PRRSV free pigs, and seeded into 96-well plates for 24 h incubation till further assays in RPMI-1640 supplemented with 10 % fetal calf serum (FBS), 2 mM L-glutamine, 0.1 mM non-essential amino acids, 1 mM sodium pyruvate and a mixture of antibiotics.

### Selection of antisense oligonucleotide sequences

Bo et al. [11] developed a database named AOBase which stores 448 AS-ONs against the transcripts of 28 different target genes, and they found that the lengths of the AS-ON in the database range from 10 nt to 22 nt, with most of the AS-ONs 20 nt long. Therefore in this study we designed and synthesized all AS-ONs with a length of 20 nt.

Alignments of over 100 sequences (from NCBI database) of each gene region were carried out, respectively. Eight AS-ONs with 20 nt length (Table 1) directed against the well-conserved regions of PRRSV with 100 % sequence similarity (i.e., 5′-UTR, NSP9, ORF5 and ORF7) were selected using RNA Structure 5.6 [12, 13]. We selected the most frequently occurring secondary structure of the target RNA with minimal overall free energy as a potential AS-ON target site. Specificity of these sequences was verified by BLAST search. The positive AS-ON control (5UP2) studied by Patel et al. [14], which is not fully complementary to the corresponding 5′UTR region of the YN-1 strain used in this study, served as a negative control. This AS-ON control was also modified to 5′-C**GT**GGCATAGAGCCAACACC-3′ here to serve as a candidate sequence for the YN-1 strain(5UP2*, see Table 1). The AS-ONs were synthesized by Shengong (Shanghai, China).

**Table 1:** List of antisense oligonulceotides used in this study

Name of AS-ON	Sequence	Target gene	Position within the target gene	GC content (%)	Overall ΔG°37	Duplex ΔG°37	Break targ. ΔG°37	Oligo-self ΔG°37	Oligo-oligo ΔG°37	Tm (°c)
5UP2	5′-CAAGGCATAGAGCCAACACC-3′	2 nt mismatching	11–30 bp	55						
5UP2*	5′-CGTGGCATAGAGCCAACACC-3′	5′UTR	11–30 bp	60	−22.3	−24.9	0	−2.6	−6.8	68.9
YN1	5′-TGGGCTGTGCCAGTGGTCAC-3′	5′UTR	52–71 bp	65	−20.6	−41.1	−15.7	−4.8	−12.6	93.1
YN2	5′-GATTGAAGGCAGTCTGGATC-3′	ORF7	234–253 bp	50	−16.1	−22.6	−5.9	−0.3	−5.7	67.2
YN3	5′-TGCAACTCCGGAAGCAAGGT-3′	5′UTR	140–159 bp	55	−17.9	−25.4	−6.8	−0.5	−8	69.7
YN4	5′-CAGCTCACATAGCGTCAAAT-3′	ORF5	131–150 bp	45	−25.2	−36.1	−9.3	−1.5	−6.4	86.7
YN5	5′--CGTACGACGGTAGATGCTCT-3′	NSP9	1266–1285 bp	55	−23.5	−25	−0.4	−0.7	−7.8	69.6
YN6	5′-CGACGACAGACACAACTGCC-3′	ORF7	215–237 bp	60	−14.1	−24.5	−9.6	−0.6	−6.4	66.4
YN7	5′- CTGGATCGACAGACAGACACA-3′	ORF7	221–240 bp	55	−15.9	−38	−20.6	−1.4	−9.5	87.5
YN8	5′-TGCAGCATCCTCACAACCGT-3′	NSP9	704–723 bp	55	−23.1	−27.8	−4.7	0	−5.9	75.3

The thermodynamic parameters are free energy changes at 37 °C in kcal/mol. “Overall ΔG°37” is the total free energy change of binding for a structured target and structured oligonucleotide. “Duplex ΔG°37” is the free energy change of duplex formation between the oligonucleotide and target. “Break Targ. ΔG°37” is the free energy cost of opening target secondary structure. “Oligo Self ΔG°37” and “Oligo-Oligo ΔG°37” are the free energy costs of opening unimolecular and bimolecular self-structure in the oligonucleotide, respectively. The Tm is the melting temperature of duplex formation in °C, not accounting for self-structure in target or oligonucleotide

The selection standards and procedures using RNAstructure were described in details by Mathews [12]. Briefly, for an oligonucleotide to tightly bind to its complementary sequence, the duplex free energy should be low and the magnitude of the cost of opening the target structure should also be minimized. The free energy of duplex formation and the oligonucleotide self-structure terms were previously shown to correlate with antisense oligonucleotide efficacy [15].

### Virus infection and transfection

The cells were trypsinized and plated in 96-well plates (Corning) at 10^4^ cells in 100 μl per well the day before PRRSV infection and AS-ON transfection. The cells were challenged with PRRSV YN1 strain (25 TCID50/well). After 90 min inoculation, the medium was removed and AS-ONs were transfected using 32 μM of AS-ONs in 100 μl of OPTI-MEM medium (Gibco) mixed with 1.5 μl/well of Lipofectamine 2000 (Invitrogen) according to the manufacturer’s instructions. Four hours post-transfection, the transfection medium was replaced with fresh full DMEM medium till further analysis. Each treatment was performed in triplicates.

To measure the life time of AS-ONs in vitro, cy-3 labeled antisense oligonucleotide 5UP2 was used in the transfection. Cy-3 fluorescence signals were observed under the fluorescence microscopy (Olympus) at various time points (1, 2, 4 and 6 h post transfection) after removal of the old medium, PBS wash and addition of fresh medium.

### Isolation of total RNA

Total RNA was isolated using the RNAiso Plus RNA isolation kit from Takara (Dalian, China), as per the manufacturer’s instructions. The RNA was quantified using the absorbance at 260 nm and the purity assessed from the ratio of absorbance at 260 and 280 nm. The RNA used for all assays had a ratio of A260/A280 greater than 1.90.

### Reverse transcription and qPCR analysis

Total RNA was isolated and subjected to reverse transcription and qPCR analysis. β-actin served as internal control. The primer pairs used in this study are: 5′-atccaggctgtgctgtcc-3′ as forward primer and 5′-gaggatcttcatgaggtagtcg-3′ as reverse primer for β-actin; 5′-gaacgctccctctgcttgc-3′ as forward primer and 5′-aaactcaacctgaaaacgttaccttc-3′ as reverse primer for ORF7. The ΔΔCt method [16] for relative quantification of gene expression was used to determine viral RNA levels. For analyzing the inhibitory effect on RNA levels, SYBR Green real-time PCR was performed. Briefly, reverse transcription was carried out using Takara PrimerScript RT reagents kit in a volume of 10 μl at 37 °C for 15 min. One microliter of reverse transcription reaction mixture was used for qPCR by using gene-specific primers and master mix from SYBR Primer Ex Taq II kit (Takara). All reactions were performed in a 25 μl reaction volume. The reaction was carried out at 95 °C for 30 s, followed by 35 cycles at 95 °C for 30 s and 60 °C for 5 s. Relative amount of PRRSV RNA were normalized to β-actin mRNA. Amplification and detection of samples were performed with the CFX96 Touch Real-Time PCR Detection System (Bio-Rad).

### Virus titration

Ten thousand Marc-145 cells were seeded into 96-well plates the day before infection and transfection. A Ten-fold serial dilution of PRRSV YN-1 strain was made with medium. 100 μl/well of each dilution was added into six wells. Ninety minutes post infection, the transfection was performed as described above. CPE was monitored using the inverted microscope over a period of 4 days post transfection. Cell number was recorded and the 50 % tissue culture infected dose (TCID50) was determined by Reed–Muench method.

### Indirect immunofluorescence staining

Sixty hours post PRRSV infection and transfection with AS-ONs, medium was removed and the MARC-145 cells were fixed with 100 μl/well of 4 % paraformaldehyde (PFA) at room temperature for 15 min followed by three washes with PBS (137 mM NaCl, 7 mM Na_2_HPO_4_, 1.5 mM KH_2_PO_4_, 2.7 mM KCl, pH 7.4), The fixed samples were permeabilized with PBS containing 0.3 % Triton X-100 for 15 min and blocked with PBS containing 1 % BSA for 2 h at 4 °C. The nuclei were stained with 5 μg/ml of Hoechst 33,342 (Life Technology) in PBS for 20 min at room temperature. Then the cells were incubated with 5 μg/ml of PRRSV antibody against N protein (encoded by ORF7) generated from mouse (VMRD, Cat. no. 080728–004) at 4 °C overnight. Cells were washed three times with PBS and incubated with Alexa Fluor 488 conjugated goat anti-mouse IgG (H + L) antibody (Proteintech, Cat. no. 861163) at 5 μg/ml for 1 h at 37 °C. After three PBS washes the cells in the 96-well plates were protected from light until further analysis by fluorescence microscopy (Olympus).

### Statistical analysis

Software SPSS 11.0 and Student’s t-test were used to determine significant differences between treatments. A p-value <0.05 was considered statistically significant. n-values represent the number of independent experiments.

## Results

### Antisense oligonucleotide design

An effective AS-ON should be selected at the RNA regions which are accessible for hybridization [17, 18]. To select potent AS-ONs, the binding energy between the AS-ONs and RNA (ΔG°37) should be as low as possible (e.g., ≤ −8 kcal/mol), whereas the energy for binding between AS-ONs should be as high as possible (e.g., ΔG°37 ≥ −1.1 kcal/mol) [19]. After analyzing some data collected from > 1000 experiments, Matveeva et al. [15] found a positive correlation between AS-ON mediated RNA knockdown and the presence of CCAC, TCCC, ACTC, GCCA and CTCT motifs in the AS-ONs, while the presence of GGGG, ACTG, AAA and TAA motifs in AS-ONs showed negative correlations. Ho et al. [19] observed strong AS-ONs effects with a minimum of 11 G or C residues in 20 nt AS-ONs.

**Fig. 1:**
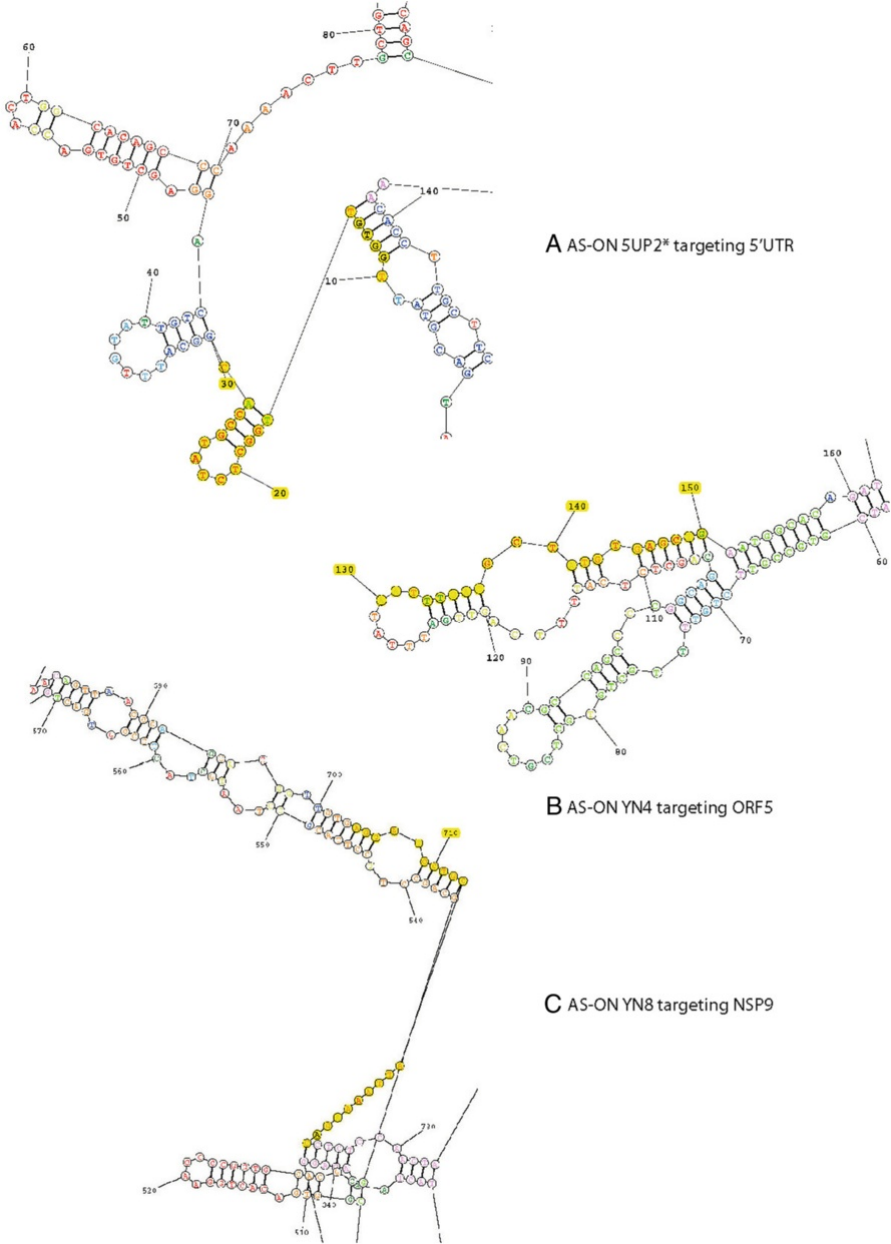
Positions of the inhibitory AS-ON hits in the PRRSV gene regions. The lowest free energy secondary structures predicted for the PRRSV RNA regions were drawn by RNAstructure. The full RNA secondary structure of PRRSV 5′UTR is shown in A, while the partial RNA secondary structures of PRRSV ORF5 and NSP9 are shown in B and C, respectively. The complementary regions between the AS-On hits (5UP2*, YN4 and YN8) and their corresponding targets were highlighted in yellow: 5UP2* targeting 5′UTR, YN4 targeting ORF5 and YN8 targeting NSP9

Nine AS-ONs were selected (Table 1) using the program OligoWalk from the package RNAstructure to calculate the binding energies of AS-ONs/AS-ONs and AS-ONs/RNA. We tried to keep the AS-ONs containing ≥ 11 G or C, or any of the positive motifs (e.g., GCCA in sequence 5UP2* and YN1) and to exclude the ones containing the negative motifs. Figure 1 illustrates the complementary sequences of the targeting gene regions for three AS-ONs showing prominent inhibitory effects on PRRSV replication and demonstrates that the local structures accessible to AS-ONs are those usually located at the terminal end, internal loops, joint sequences, hairpins and bulges of ≥10 consecutive nucleotides, as previously reported by Kretschmer-Kaxemi et al. [20].

### Life time of antisense oligonucleotides in in vitro cell culture

**Fig. 2:**
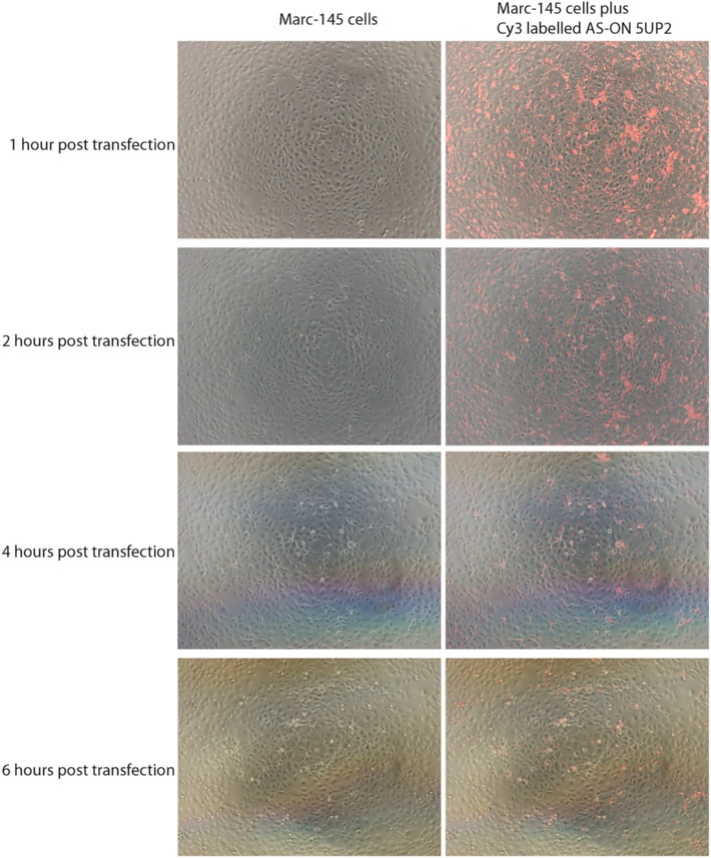
AS-ON was present in cell culture six hours post transfection. Cy3-labelled antisense oligonucleotide 5UP2 (32 μM) was used in the transfection in Marc-145 cells. The fluorescent signals were recorded with fluorescence microscopy (Olympus) at various time points post transfection. Cells are shown to the left and the merged images (cells with fluorescent oligonucleotides) to the right. Although fluorescent signals decreased over the time, we could still clearly observe the signals even six hours post transfection, indicating that the antisense oligonucleotide persist in Marc-145 cells for sufficiently long time periods to induce the degradation of viral RNA and to inhibit PRRSV replication

To investigate how long AS-ONs are present in an in vitro cell culture system, the Cy-3 labelled antisense oligonucleotide 5UP2 was transfected into MARC-145 cells. At different time points (1, 2 , 4 and 6 h) post transfection, the medium was removed, the cells were washed with PBS, fresh medium was added into the wells, and cells were imaged. (Fig. 2). We found that the fluorescent signal from Cy-3 labelled antisense oligonucleotides was still detectable 6 h post transfection, although the signal decreased through the observation time period, demonstrating that the antisense oligonucleotide is not degraded prior to PRRSV mRNA transcription and thus can target to the viral mRNA for its degradation.

### Inhibition of CPE by transfection with antisense oligonucleotides

**Fig. 3:**
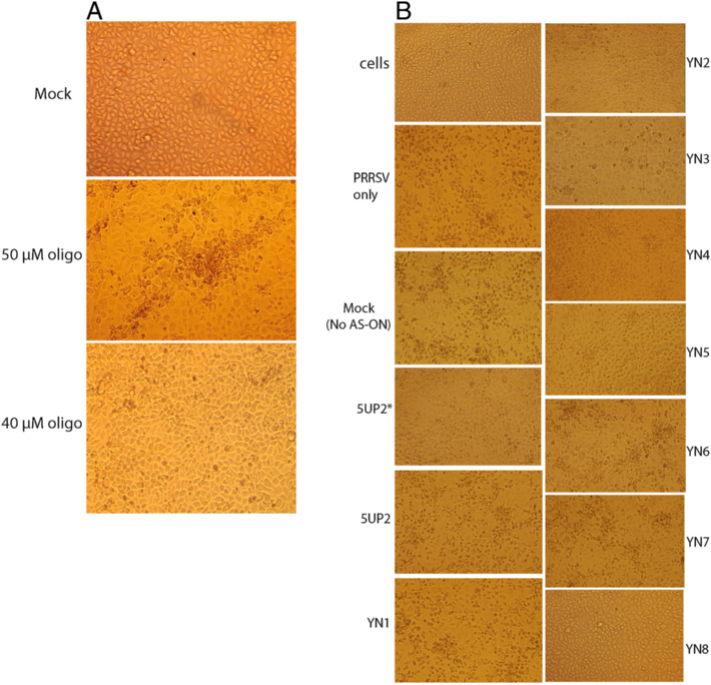
Cytopathic effect (CPE) analysis of porcine reproductive and respiratory syndrome virus (PRRSV) in MARC-145 cells transfected with AS-ONs. **a**. Comparing with the mock treatment, transfection with 50 μM antisense oligonucleotide 5UP2 showed significant cytotoxicity, while transfection with 40 μM did not. **b**. Four out of nine selected antisense oligonucleotides demonstrated significant (5UP2* and YN8) or moderate (YN4 and YN5) protection from challenge with PRRSV YN-1 strain. Pictures were taken 72 h post infection with a Nikon E5400 camera mounted on an inverted microscope (Nikon TS100)

To investigate whether AS-ONs can protect MARC-145 cells from the cytopathic effect (CPE) induced by PRRSV, cells were transfected with AS-ONs one and half hours post challenge with 25 TCID50 PRRSV per well. Treatments of no transfection, mock (Lipofectamin 2000 only, without any antisense sequence) and the 2 nt mismatching oligo 5UP2 served as negative controls. CPE was recorded daily. An image for each treatment was acquired 72 h post transfection and is shown in Fig. 3. Significant cytotoxic effects were observed neither in the mock well nor in the transfection well with 40 μM of 5UP2, but in the transfection well with 50 μM of 5UP2 (Fig. 3a), indicating that 40 μM of AS-ONs is a safe dose for the cells. Therefore the working concentration (32 μM) of AS-ONs in this study would not be toxic to the cells, ruling out the possibility of inhibitory effects due to the toxicity of different AS-ONs. In Fig. 3b, compared with the normal cells (neither PRRSV inoculation nor transfection was applied), the cells in the negative controls aggregated, rounded up and disintegrated. We found that cells transfected with 5UP2*, YN4, YN5 and YN8 manifested overtly less CPE. Other AS-ON-treated cells and the mock demonstrated the same typical PRRSV-induced CPE as cells infected only with virus (PRRSV only), which became pycnotic and detached from the monolayer, demonstrating that those antisense oligonucleotides do not show any obvious interference with viral replication.

### Reduction of viral RNA levels by transfection with antisense oligonucleotides

**Fig. 4:**
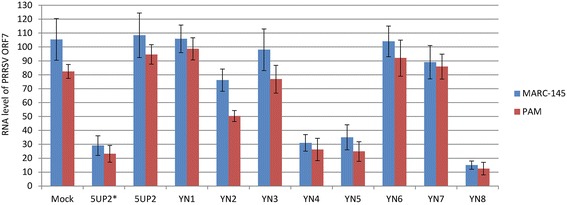
Viral ORF7 RNA level was reduced by transfection with selected antisense oligonucleotides in both Marc-145 and PAM cells. Four of the nine selected antisense oligonucleotides (5UP2*, YN8, YN4 and YN5) significantly inhibited the RNA level of gene ORF7. After virus challenge and transfection with AS-ONs under the same conditions as in the CPE analysis experiment, total RNA was isolated and subjected to reverse transcription and qPCR analysis. The ΔΔCt method for relative quantification of gene expression was used to determine viral RNA levels. The Y-axis shows the relative RNA levels of the ORF7 gene for each treatment after normalization to the non-transfected reference sample using β-actin as internal control. The values shown are means of three independent experiments

To investigate whether AS-ONs inhibit PRRSV replication in MARC-145 cells and PAM, MARC-145 cells and PAM were transfected by the selected AS-ONs. Non-transfected MARC-145 cells and PAM challenged with PRRSV were used as neutral control. Mock and transfection with 5UP2 were applied as controls for nonspecific inhibition. When normalized to β-actin mRNA, similarly in both Marc-145 and PAM, the ORF7 RNA level in the cells transfected with AS-ONs YN8, 5UP2*, YN4 and YN5 were reduced by 85, 75, 70 and 70 %, respectively (Fig. 4). There was no significant inhibition in cells transfected with the other five AS-ONs and the 2 nt mismatching oligo 5UP2, suggesting that the reductions in ORF7 RNA did not result from nonspecific inhibition or toxicity.

### Reduction of viral titer by transfection with antisense oligonucleotides

**Fig. 5:**
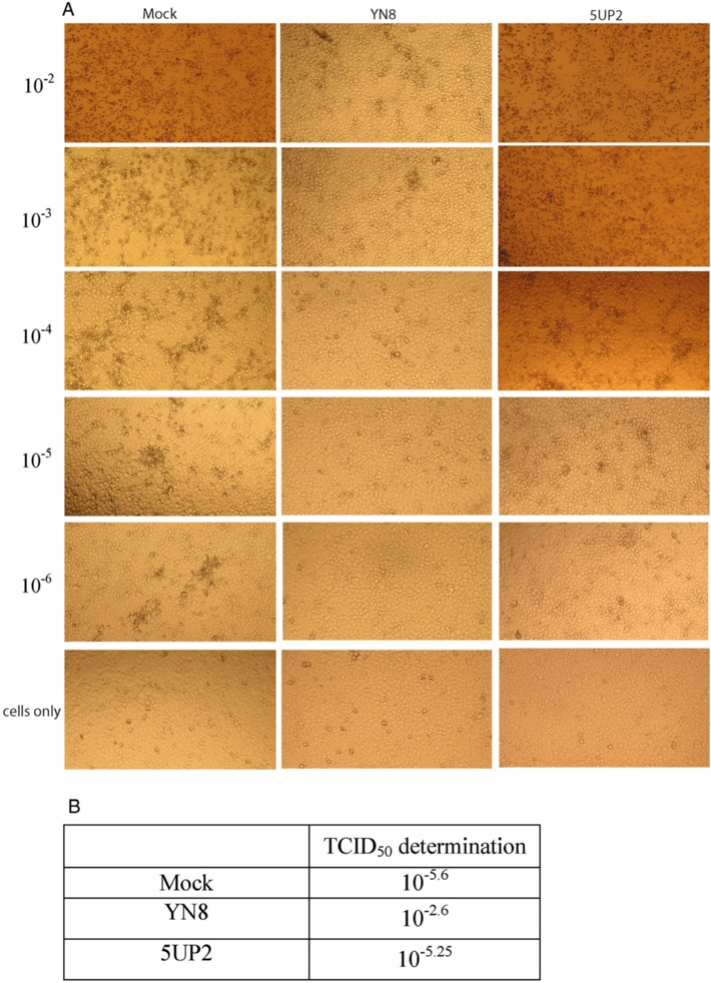
The antisense oligonucleotide sequence YN8 protected PAM from cytopathic effects and reduced the viral titer by 1000 fold. Pulmonary alveolar macrophages (PAM) (10 000 cells per well) were seeded into 96-well plates the day before infection and transfection. Ten-fold serial dilution of PRRSV YN-1 strain was made with medium. 100 μl/well of each dilution was added into six wells. Ninety minutes post infection, the transfection was performed. CPE was monitored using the inverted microscope over a period of 4 days post transfection. The 50 % tissue culture infected dose (TCID50) was determined by Reed–Muench method. Comparing to the mock and 2-nt mismatch 5UP2control groups, transfection with antisense oligonucleotide sequence YN8 significantly protected the PAM from cytopathic effects (**a**) and reduced the viral titer by 1000-fold (**b**)

In order to further determine the level of inhibition, CPE was monitored until 4 days post virus infection and transfection. Viral titers were measured by TCID50 assay. We found that compared to the mock and 2-nt mismatch 5UP2 control groups, transfection with antisense oligonucleotide sequence YN8 significantly protected Marc-145 cells from cytopathic effects (Fig. 5a) and reduced the viral titer by 1000-fold (Fig. 5b).

### Abrogation of viral protein level by transfection with antisense oligonucleotides

**Fig. 6:**
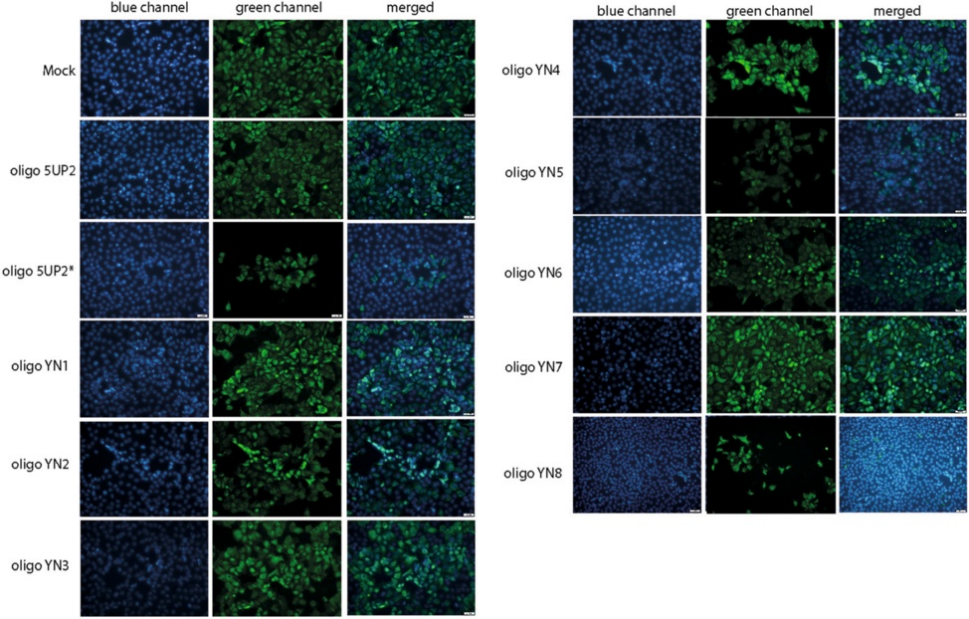
Indirect immunofluorescence detection of PRRSV in MARC-145 cells transfected with or without AS-ONs. Transfection was performed 90 min post infection with PRRSV YN-1 strain (25 TCID50). Cells were fixed 60 h post transfection, reacted against anti-N monoclonal antibody (MAb) and then incubated with fluoresce in Alexa Fluo488 conjugated secondary antibody. Blue color stains nuclei to show the total number of cells. The green color indicates the expression of N protein in MARC-145 cells. The images of the same treatment from the two channels were merged. Treatment with YN8, 5UP2*, YN4 and YN5 resulted in reduction of virus replication, while 5UP2 and the other AS-ONs did not appear to have obvious effects under identical conditions

To investigate the effect of AS-ONs on inhibiting the expression of viral protein, indirect immunofluorescence assays were performed with anti-N protein mAb (encoded by gene ORF7) 60 h post transfection. Indeed, fewer fluorescing cells were seen in the monolayers treated with YN8 and 5UP2* than in the other monolayers, while transfection with YN4 and YN5 mildly reduced the viral N protein level. In contrast, the infected cells in wells pre-transfected with YN1, YN2, YN3, YN6 and YN7 had equivalent or slightly less N protein-positive cells compared to that of controls (i.e., mock and transfection with the 2 nt mismatching oligo 5UP2, Fig. 6).

## Discussion

PRRSV is recognized as one of the most important viruses for swine industry. It can persist in pigs for quite long time after initial infection, and infected animals with persistent infection can shed infectious virus for several months. Many scientists have been putting a lot of efforts into developing various shRNA or siRNA methods to suppress PRRSV replication in vitro and in vivo [21–27], and only few studies have used antisense technology [14, 28]. However, it is well known that antisense technology has outperformed siRNAs to some extent [29, 30], as it is easy and quick to design an antisense against a gene target, to produce the antisense sequence effectively in scale using standard equipment and procedures at lower cost and its modified derivatives that offer greater stability and efficacy, to deliver AS-ONs into cells.

Therefore, in this study we aimed to design AS-ONs targeting PRRSV regions of 5′UTR, ORF5, ORF7 and NSP9, and to test the inhibitory effects of these AS-ONs without any chemical modifications in cell culture. Under the conditions of this study, four out of the nine selected AS-ONs showed prominent inhibitory effectson PRRSV replication. The reasons for the ineffectiveness of the other AS-ONs are unclear, but could include the inaccessibility of the target sequence, or difficulties in secondary structure prediction of viral RNA. Alternatively, successful AS-ON/target RNA duplexing might not necessarily affect PRRSV replication. However, compared with the reported hit rates (approx. 10 % [31], 4/34 [32] or 4/12 [14]), we achieved higher ‘success rate’ (44 %). As shown in Table 1, the four effective AS-ONs have the lowest value of overall ΔG°37, indicating that the binding between the antisense oligonucleotide and its corresponding target sequence might be very stable. However, no obvious conclusions could be drawn regarding the efficacy and specific motifs or GC content in the AS-ON sequences. Larger sets of AS-ONs should be tested to obtain more statistically significant data.

Blasting the four inhibitory AS-ONs revealed that these sequences are highly conserved and hit more than 100 PRRSV strains of the Northern American type, with most of them reported from China. This suggests that that these AS-ONs should inhibit the replication of Northern American strains of PRRSV prevalent in China, not only limited to the YN-1 stain which was isolated from the southwest of China, and thus can be considered as potential drug candidates for use in PRRSV control.

## Conclusion

In conclusion, this study demonstrates that DNA-based short antisense oligonucleotides can effectively inhibit PRRSV replication in MARC-145 cells and in PAMs. The experimental results which are based on the CPE experiments, measurement of the viral ORF7 RNA level and indirect fluorescence staining, demonstrated that MARC-145 cells transfected with AS-ON 5UP2* (targeting 5′-UTR) and YN8 (targeting NSP9) showed a significant decrease in virus replication when compared to the 2 nucleotide mismatching negative control (AS-ON 5UP2), indicating the specificity of the antisense oligonucleotides; while transfection with YN4 or YN5 also inhibited the PRRSV replication to some extents. It infers that 5′-UTR or NSP9 might be one of the optimal viral targets to design AS-ONs. However, the natural phosphodiester oligodeoxynucleotides (DNAs) used in this study could be relatively quickly degraded by nucleases. When it comes to the actual in vivo application of AS-ONs in therapeutic control of PRRSV, we plan to add some locked nucleic acid (LNA) modifications to the four promising AS-ON sequences for further studies both in vitro and in vivo, in order to increase the stability of AS-ONs and the cellular uptake, to enhance the hybridization affinity towards target mRNA and to reduce the toxicity, to investigate the techniques and efficiency of delivery, to study the temporary effects and to address the issue of emergence of escape mutants.

## Abbreviations

PRRSV: Porcine reproductive and respiratory syndrome virus
PRRS: Porcine reproductive and respiratory syndrome
AS-ONs: Antisense oligonucleotides
UTR: Untranslated region
NSP: Non-structural protein
ORF: Open reading frame
CPE: Cytopathic effect
qPCR: Quantitative PCR
FBS: Fetal bovine serum
PAM: Pulmonary alveolar macrophages
TCID50: 50 % Tissue Culture Infective Dose
hr: Hour
hpi: Hours post infection
LNA: Locked nuclei acid

## References

[1] Pejsak Z, Markowska-Daniel I (1997). Losses due to porcine reproductive and respiratory syndrome in a large swine farm. Comp Immunol Microbiol Infect Dis.

[2] Meulenberg Janneke JM (2000). PRRSV, the virus. Vet Res.

[3] Dea S, Gagnon CA, Mardassi H, Pirzadeh B, Rogan D (2000). Current knowledge on the structural proteins of porcine reproductive and respiratory syndrome (PRRS) virus: comparison of the North American and European isolates. Arch Virol.

[4] Wang FX, Wen YJ, Yang BC, Liu Z, Shi XC, Leng X (2012). Role of non-structural protein 2 in the regulation of the replication of the porcine reproductive and respiratory syndrome virus in MARC-145 cells: effect of gene silencing and over expression. Vet Microbiol.

[5] Verheije MH, Olsthoorn RC, Kroese MV, Rottier PJ, Meulenberg JJ (2002). Kissing interaction between 3′ noncoding and coding sequences is essential for porcine arterivirus RNA replication. J Virol.

[6] Verheije MH, Kroese MV, Rottier PJ, Meulenberg JJ (2001). Viable porcine arteriviruses with deletions proximal to the 3′ end of the genome. J Gen Virol.

[7] Kurreck J (2003). Antisense technologies Improvement through novel chemical modification. Eur J Biochem.

[8] Crooke ST (2004). Progress in antisense technology. Annu Rev Med.

[9] Gleave ME, Monia BP (2005). Antisense therapy for cancer. Nat Rev Cancer.

[10] Tian K, Yu X, Zhao T, Feng Y, Cao Z, Wang C (2007). Emergence of fatal PRRSV variants: unparalleled outbreaks of atypical PRRS in China and molecular dissection of the unique hallmark. PLoS One.

[11] Bo X, Lou S, Sun D, Shu W, Yang J, Wang S (2006). Selection of antisense oligonucleotides based on multiple predicted target mRNA structures. BMC Bioinformatics.

[12] Mathews DH (2006). RNA secondary structure analysis using RNAstructure. Curr Protoc Bioinformatics.

[13] Reuter JS, Mathews DH (2010). RNAstructure: software for RNA secondary structure prediction and analysis. BMC Bioinformatics.

[14] Patel D, Opriessnig T, Stein DA, Halbur PG, Meng XJ, Iversen PL (2008). Peptide-conjugated morpholino oligomers inhibit porcine reproductive and respiratory syndrome virus replication. Antiviral Res.

[15] Matveeva OV, Tsodikov AD, Giddings M, Freier SM, Wyatt JR, Spiridonov AN (2000). Identification of sequence motifs in oligonucleotides whose presence is correlated with antisense activity. Nucleic Acids Res.

[16] Pfaffl MW (1999). A new mathematical model for relative quantification in real time RT-PCR, Nucleic Acids Research, 29, 2002–2007, 2001ABI PRISM 7700 Sequence Detection System.

[17] Ho SP, Bao Y, Lesher T, Malhotra R, Ma LY, Fluharty SJ (1998). Mapping of RNA accessible sites for antisense experiments with oligonucleotide libraries. Nat Biotechnol.

[18] Ding Y, Lawrence CE (2001). Statistical prediction of single-stranded regions in RNA secondary structure and application to predict effective antisense target sites and beyond. Nucleic Acids Res.

[19] Ho SP, Britton DH, Stone BA, Behrens DL, Leffet LM, Hobbs FW (1996). Potent antisense oligonucleotides to the human multidrug resistance-1mRNA are rationally selected by mapping RNA-accessible sites with oligonucleotide libraries. Nucleic Acids Res.

[20] Kretschmer-Kaxemi Far R, Nedbal W, Sczakiel G (2001). Concepts to automate the theoretical design of effective antisense oligonucleotides. Bioinformatics.

[21] Huang J, Jiang P, Li Y, Xu J, Jiang W, Wang X (2006). Inhibition of porcine reproductive and respiratory syndrome virus replication by short hairpin RNA in MARC-145 cells. Vet Microbiol.

[22] He YX, Hua RH, Zhou YJ, Qiu HJ, Tong GZ (2007). Interference of porcine reproductive and respiratory syndrome virus replication on MARC-145 cells using DNA-based short interfering RNAs. Antiviral Res.

[23] Li G, Huang J, Jiang P, Li Y, Jiang W, Wang X (2007). Suppression of porcine reproductive and respiratory syndrome virus replication in MARC-145 cells by shRNA targeting ORF1 region. Virus Genes.

[24] Bao Y, Guo Y, Zhang L, Zhao Z, Li N (2012). Inhibition of porcine reproductive and respiratory syndrome virus replication by RNA interference in MARC-145 cells. Mol Biol Rep.

[25] Yang M, Xiang Q, Zhang X, Li X, Sylla S, Ding Z (2014). RNA interference targeting nucleocapsid protein inhibits porcine reproductive and respiratory syndrome virus replication in Marc-145 cells. J Microbiol.

[26] Luo B, Ju S, Wang B, Rui R (2013). A possible strategy to produce pigs resistant to porcine reproductive and respiratory syndrome virus. Antiviral Res.

[27] Li L, Li Q, Bao Y, Li J, Chen Z, Yu X (2014). RNAi-based inhibition of porcine reproductive and respiratory syndrome virus replication in transgenic pigs. J Biotechnol.

[28] Han X, Fan S, Patel D, Zhang YJ (2009). Enhanced inhibition of porcine reproductive and respiratory syndrome virus replication by combination of morpholino oligomers. Antiviral Res.

[29] Dove A (2002). Antisense and sensibility. Nat Biotechnol.

[30] Malik R, Roy I (2008). Design and development of antisense drugs. Expert Opin Drug Discov.

[31] Gewirtz AM, Sokol DL, Ratajczak MZ (1998). Nucleic acid therapeutics: state of the art and future prospects. Blood.

[32] Myers KJ, Dean NM (2000). Sensible use of antisense: how to use oligonucleotides as research tools. Trends Pharmacol Sci.

